# Interleukin 21 inhibits cancer-mediated FOXP3 induction in naïve human CD4 T cells

**DOI:** 10.1007/s00262-017-1970-6

**Published:** 2017-02-27

**Authors:** Vinodh Kannappan, Kate Butcher, Malgorzata Trela, Iain Nicholl, Weiguang Wang, Kesley Attridge

**Affiliations:** 1 Research Institute in Healthcare Science, University of Wolverhampton, Wolverhampton, UK; 2 School of Life and Health Sciences, MB Building, Aston University, Aston Triangle, B4 7ET Birmingham, UK

**Keywords:** Regulatory T cells, FOXP3, Immunosuppression, IL-21, Immunotherapy, Anti-tumour immunity

## Abstract

IL-21 is known to promote anti-tumour immunity due to its ability to promote T cell responses and counteract Treg-mediated suppression. It has also been shown to limit Treg frequencies during tumour-antigen stimulations. However, whether this represents inhibition of FOXP3 induction in naïve CD4 T cells or curtailed expansion of natural Treg remains unclear. Moreover, whether this effect is maintained in an environment of tumour-derived immunosuppressive factors is not known. Here, we show that in the context of a number of cancers, naïve CD45RA+ CD4 T cells are induced to express high levels of FOXP3, and that FOXP3 expression correlates with inhibition of T cell proliferation. FOXP3 expression was most potently induced by tumours secreting higher levels of total and active TGFβ1 and this induction could be potently counteracted with IL-21, restoring T cell proliferation. We conclude that Treg induction in naïve T cells is a common phenomenon amongst a number of different cancers and that the ability of IL-21 to counteract this effect is further evidence of its promise in cancer therapy.

## Introduction

Immunosuppressive FOXP3+ regulatory T cells (Treg) are known to accumulate within the tumour microenvironment in a variety of cancers and are associated with deficiencies in effector T cell responses and poorer outcomes [1]. In gliomas, for example, Treg cells are found within tumour tissue and their accumulation correlates with advanced tumour stages [2–4]. The ratio of Treg cells to effector cell populations, such as CD8 T cells, also appears to be prognostically significant, with high CD8 T cell to Treg ratios being linked with more favourable outcomes in ovarian cancers [5]. Moreover, in animal models of a number of different cancers, it has long been known that Treg cell depletion restores anti-tumour immunity and promotes tumour rejection [6–9]. Treg, therefore, represent a barrier to effective natural anti-tumour immunity, but whether intra-tumoural Treg are recruited from existing peripheral populations or are induced to differentiate from naive CD4 T cells within the tumour microenvironment remains unclear. Both mechanisms likely play an important role and interestingly, both in vitro and in vivo CD4 T cell stimulations with tumour antigens have been shown to induce Treg differentiation, even in the absence of other tumour-derived immunosuppressive factors [10, 11]. This has important implications not only within the tumour microenvironment, but also for anti-tumour immunotherapeutic strategies, such as cancer vaccines and engineered chimeric antigen receptor (CAR) T cells, where Treg induction would be counter-productive and could significantly limit their potential.

In recent years, a number of cytokines have been found to inhibit regulatory T cell homeostasis and function, and thus may represent a route to alleviate Treg-mediated immunosuppression in cancer. In this respect, IL-21 has shown significant potential due to its ability to counteract the Treg-mediated inhibition of CD4 T cell responses in both murine [12, 13] and human studies [14]. This effect was found to be mediated indirectly by signaling to conventional T cells [13, 15], and was associated primarily with diminished production of IL-2, a cytokine known to be essential for Treg survival [15]. IL-21 has also been demonstrated to boost CD4 T cell responses via the upregulation of the co-stimulatory ligand CD86 on antigen-presenting cells [16], and is essential for the long-term maintenance of CD8 T cell function [17].

In the context of cancer, a crucial finding has been that IL-21 reduces the frequency of human FOXP3+ Treg cells by greater than 10-fold during tumour antigen stimulations [10]. However, whether this represents inhibition of FOXP3 induction in naive CD4 T cells or curtailed expansion of the natural Treg pool is not entirely clear. Moreover, it is not yet known whether IL-21 can maintain this effect in the presence of immunosuppressive tumour-derived factors, and if so, whether this is broadly applicable across tumour-types. In this study, we have investigated the ability of IL-21 to inhibit FOXP3 induction in purified naïve CD4 T cells in the context of culture supernatants from a number of cancer cell lines. We show that, in the majority of cases, tumour-derived factors markedly promote Treg induction, particularly for those derived from colon cancers. Colon cancer cells also produced the highest levels of total and active TGFβ1, blockade of which prevented Treg induction. Similarly, IL-21 was able to inhibit tumour-mediated FOXP3 induction in naïve CD4 T cells and restore their proliferative capacity. Thus, in addition to its documented ability to prevent de novo Treg generation [18–20], IL-21 is able to maintain this effect in the context of tumour-derived milieus that actively promote FOXP3 expression.

## Materials and methods

### Ethics

Informed consent was obtained from all blood donors and the study was approved by an institutional ethics committee.

### Cell lines

Five cell lines were used representing cancers of the colon (RKO, HCT116), lung (A549), liver (PLC/PRF-5) and brain (U87 MG). All cells were cultured in Dulbecco’s modified eagle’s medium (DMEM; Lonza) supplemented with 10% foetal bovine serum (Thermo Scientific), 2 mM l-Glutamine, 50 units/mL Penicillin and 50 g/mL Streptomycin. Cells were incubated at 37 °C in a humidified atmosphere (5% CO_2_). Culture supernatants were isolated from adherent cultures at days 3 or 4 and filtered through a cell strainer to remove cellular contamination prior to use in assays.

### T cell isolation and culture

PBMCs were isolated from human peripheral blood by density gradient centrifugation over Histopaque (Sigma). Magnetic separation (StemCell Technologies) was then used to isolate naïve CD45RA+ CD4 T cells. Purified cells were cultured at 2.5 × 10^4^ per well at a 1:1 ratio with CD3/CD28 human T-activator beads (Gibco) for 5 days. Where indicated, cancer cell line culture supernatants were added as a fraction of the total volume of culture media (12.5, 25 or 50%). For TGFβ blockade experiments, an anti-TGFβ antibody (1D11; R&D Systems) was used at 50 μg/mL. Where indicated, human recombinant IL-21 (Peprotech) was used at 200 ng/mL. At day 5, cells were harvested and analysed by flow cytometry. Cell counts are expressed as a percentage, with the cell count obtained for naïve T cells stimulated alone set at 100% (the maximal response).

### Cultured suppressor assay

Purified naïve CD45RA+ CD4 T cells were cultured at 2.5 × 10^4^ per well at a 1:1 ratio with CD3/CD28 human T-activator beads (Gibco) for 5 days alone, in the presence of either 50% of the indicated cancer cell line supernatants or 200 ng/mL IL-21 (Peprotech), or with both. At day 5 stimulator beads were removed magnetically and cells were washed 3 times with PBS before culture (as suppressors) with 2.5 × 10^4^ freshly purified CFSE-labelled naïve CD4+ T cells from the same donor at a 2:1 ratio (cultured suppressor T cells to freshly isolated naïve T cells). CD3/CD28 human T-activator beads (Gibco) were again used at a 1:1 ratio. At day 5 cells were harvested and analysed by flow cytometry. Cell counts are expressed as a percentage, with the cell count obtained for CFSE-labelled naïve T cells incubated with control IL-21-stimulated suppressor T cells set at 100% (the maximal response).

### Flow cytometry

Cells were stained with monoclonal antibodies against CD4 (RPA-T4; eBioscience), FOXP3 (236A/E7; eBioscience), CD25 (M-A251; BD Biosciences), CTLA-4 (BN13; BD Biosciences) and TGFβ (TW4-9E7; BD Biosciences). For intracellular staining, cells were fixed and permeabilised according to the manufacturer’s instructions (eBioscience). Stained cells were acquired using an Accuri C6 flow cytometer (BD Biosciences) and data analysed using FlowJo software (TreeStar). Statistical analyses were performed using a two-tailed paired *t* test with a 95% confidence interval. For the inverse correlation of FOXP3 expression with T cell proliferation a simple linear regression analysis was performed.

### TGFβ1 ELISA

Active TGFβ1 levels were determined using a sandwich ELISA according to the manufacturer’s instructions (eBioscience) and were derived from a standard curve of known TGFβ1 concentrations. To assay total TGFβ1 levels, culture supernatants were incubated with 1N HCl for 20 min before neutralization with 1N NaOH prior to the assay being performed. ELISA plates were read at 450 nm and absorbances for ELISA buffer alone controls were subtracted prior to analysis. Statistical analyses were performed using a two-tailed unpaired *t* test with a 95% confidence interval.

## Results

**Fig. 1:**
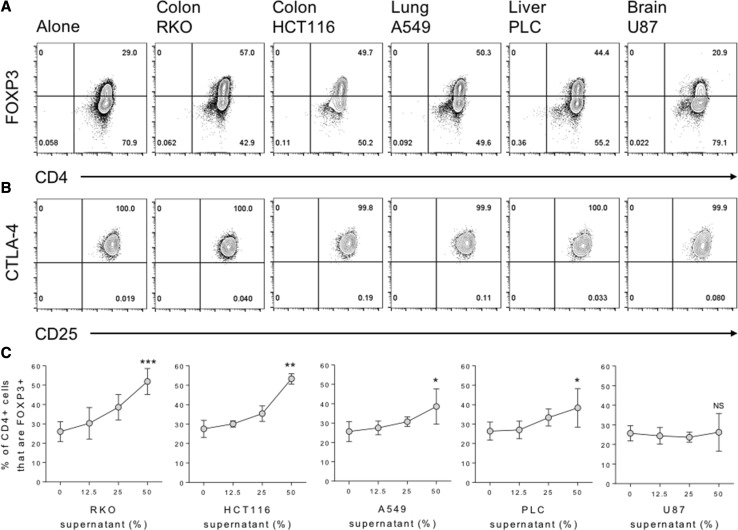
Cancer-mediated induction of a Treg phenotype in naïve human CD4 T cells. **a** 2.5 × 10^4^ CD45RA+ CD4+ T cells from human peripheral blood were stimulated with anti-CD3/28 antibody-coated beads (1:1 ratio) alone or in the presence of 50% culture supernatant from the indicated cancer cell lines. After 5 days cells were stained with CD4 PE-Cy7, FOXP3 APC, CTLA-4 PE and CD25 FITC for acquisition by flow cytometry. **b** Contour plots show expression of CD25 and CTLA-4 by gated CD4+ FOXP3+ cells. **c** Percentage of harvested CD4+ cells expressing FOXP3 across a titration of the indicated cancer supernatants. Data are representative of 4 independent experiments. *P < 0.05; **P < 0.01; ***P < 0.001

**Fig. 2:**
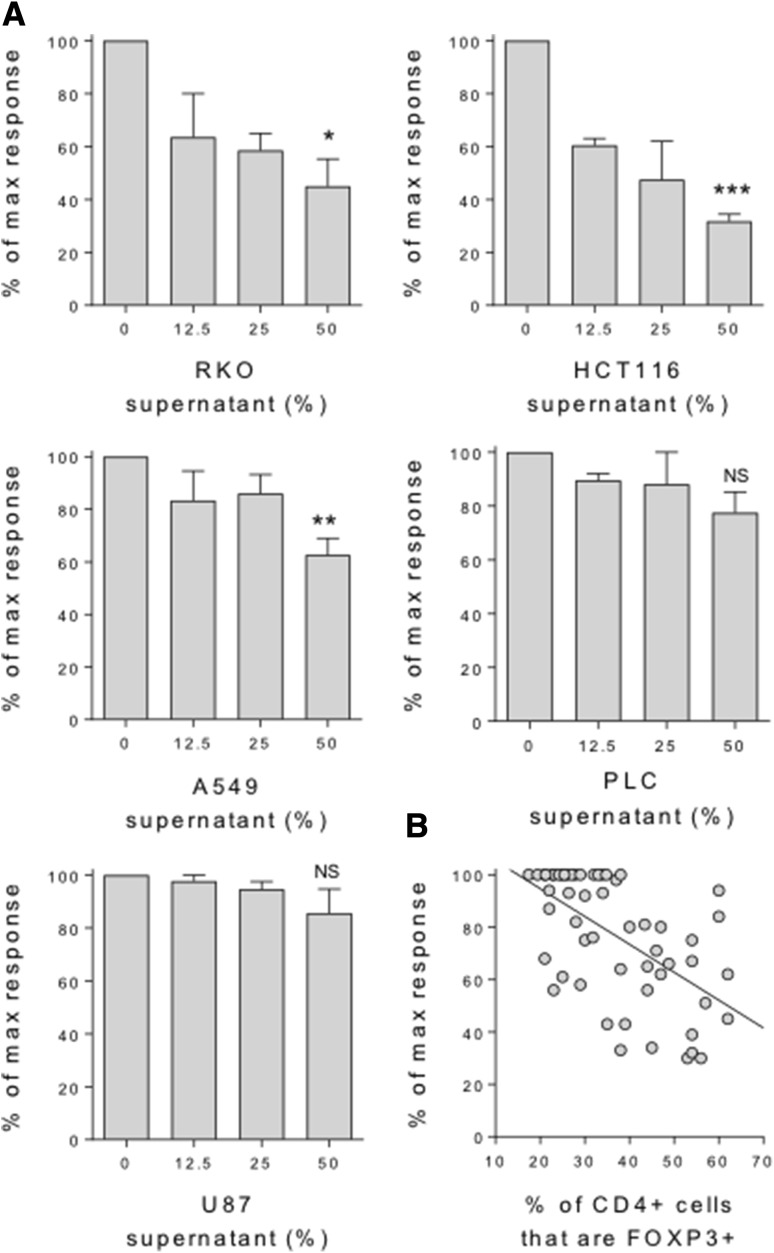
Induction of FOXP3 correlates with inhibition of the naïve T cell response. **a** 2.5 × 10^4^ CD45RA+ CD4+ T cells from human peripheral blood were stimulated with anti-CD3/28 antibody-coated beads (1:1 ratio) alone or in the presence of a titration of culture supernatant from the indicated cancer cell lines. After 5 days cells were stained with CD4 PE-Cy7 and FOXP3 APC for acquisition by flow cytometry. Histograms show CD4+ cell counts expressed as a proportion of the control naïve T cell count in the absence of cancer supernatant. **b** Inverse correlation between % FOXP3+ cells and T cell proliferation across all naïve T cell cultures with cancer supernatants (*P* < 0.0001). Data are representative of 4 independent experiments. *P < 0.05; **P < 0.01; ***P < 0.001

To determine whether cancer cells are capable of directly inducing FOXP3 expression in naïve T cells, we purified CD45RA+ CD4 T cells from human peripheral blood and stimulated them for 5 days with anti-CD3/28 antibody-coated beads, in the presence or absence of culture supernatants from five cancer cell lines representing tumours of the colon, lung, liver and brain. We observed greatly enhanced FOXP3 induction in the presence of supernatants from colon, lung and liver, but not brain cancer cells over that observed in their absence (Fig. 1a). These FOXP3 + cells also expressed other Treg phenotypic hallmarks, including high levels of CD25 and the inhibitory receptor CTLA-4 (Fig. 1b). FOXP3 induction was titratable, in that increasing the dose of cancer supernatant from 12.5 to 25%, and again to 50% of the total culture media leads to greater increases in FOXP3 expression in the naïve T cells, particularly for supernatants representing colon cancers (Fig. 1c). In these same cultures T cell proliferation was also inhibited, in a dose-dependent manner, by supernatants representing colon and lung, but not liver and brain cancers (Fig. 2a). Moreover, a significant inverse correlation was observed between FOXP3 expression and T cell proliferation, such that increasing FOXP3 induction correlated with inhibition of the T cell response (Fig. 2b).

**Fig. 3:**
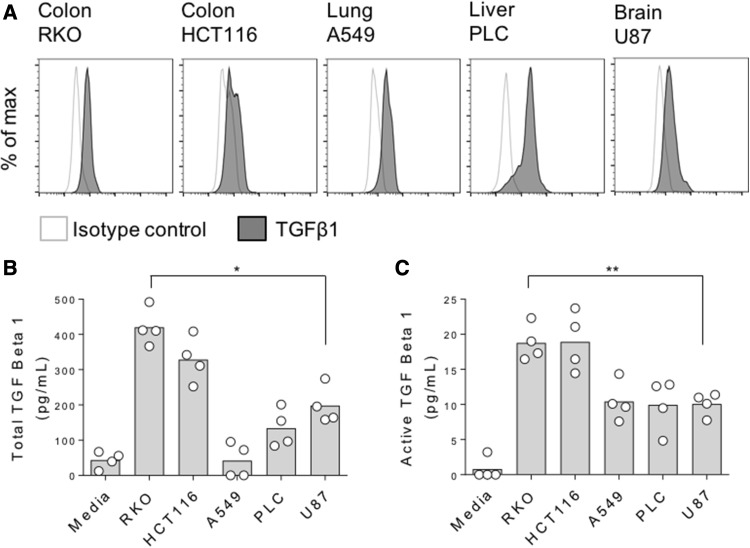
Induction of FOXP3 in naïve T cells is linked with TGFβ1 secretion by cancer cells. **a** Indicated cancer cell lines were stained for intracellular TGFβ1 expression and acquired by flow cytometry. **b** Total and **c** active TGFβ1 concentrations for culture supernatants from the indicated cancer cell lines or media alone controls. Data are representative of 3–4 independent experiments. *P < 0.05; **P < 0.01

**Fig. 4:**
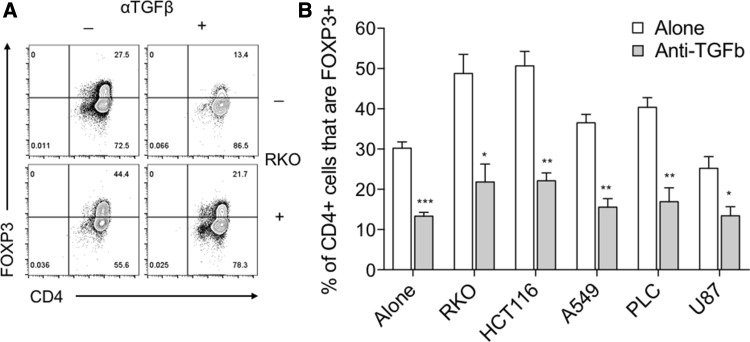
TGFβ1 blockade prevents cancer-mediated FOXP3 induction. **a** 2.5 × 10^4^ CD45RA+ CD4+ T cells from human peripheral blood were stimulated with anti-CD3/28 antibody-coated beads (1:1 ratio) alone, with either 50% cancer cell culture supernatant or 50 μg/mL blocking anti-TGFβ antibody, or both. After 5 days cells were stained with FOXP3 APC and CD4 PE-Cy7 and acquired by flow cytometry. Representative contour plots are shown for cells cultured with RKO supernatant. **b** Histogram shows collated FOXP3+ CD4+ frequencies for these cultures. Data are representative of 3 independent experiments. *P < 0.05; **P < 0.01; ***P < 0.001

As TGFβ1 has been shown to induce FOXP3 expression in naïve T cells [21, 22], we next determined whether our cancer cell lines expressed TGFβ1 by flow cytometry. Even in the absence of stimulation, basal TGFβ1 expression was observed in all five cell lines (Fig. 3a). To assess whether these cells subsequently secreted TGFβ1, we performed ELISAs assaying both active and, after acid-based release from its latent complex, total TGF β1. By this method, we found the highest concentrations of active and total TGFβ1 to be present in supernatants that induced the greatest expression levels of FOXP3 expression in naïve T cells (Fig. 3b, c). To confirm this role for TGFβ1, we repeated our naïve T cell stimulations with cancer cell supernatants, in the context of TGFβ blockade mediated by a blocking anti-TGFβ antibody. In these assays, anti-TGFβ inhibited both the baseline FOXP3 induction observed in naïve T cells cultured alone, and that seen as a result of a 50% dose of cancer cell supernatants (Fig. 4a, b).

**Fig. 5:**
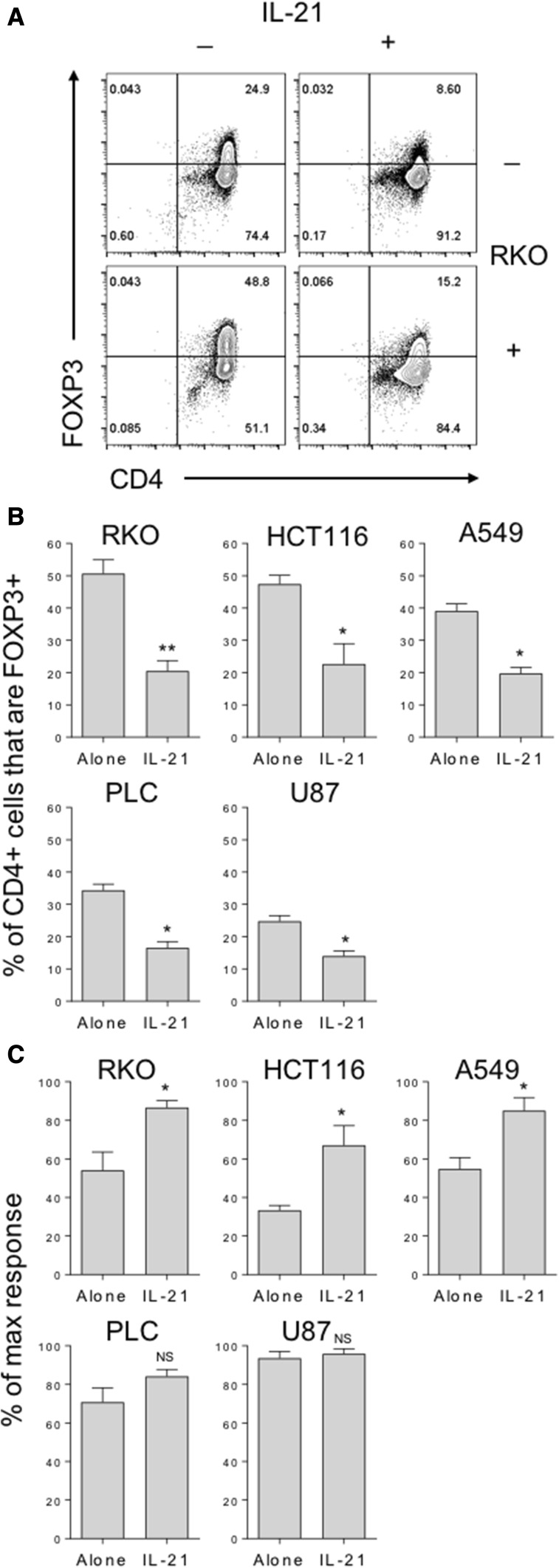
IL-21 counteracts cancer-mediated FOXP3 induction and suppression of naïve T cell proliferation. **a** 2.5 × 10^4^ CD45RA+ CD4+ T cells from human peripheral blood were stimulated with anti-CD3/28 antibody-coated beads (1:1 ratio) alone, with either 50% culture supernatant from the indicated cell lines or 200 ng/mL IL-21, or both. After 5 days cells were stained with FOXP3 APC and CD4 PE-Cy7 and acquired by flow cytometry. **b** Histograms show collated FOXP3+ CD4+ frequencies and **c** T cell proliferation data for these cultures. Data are representative of 4 independent experiments. *P < 0.05; **P < 0.01

To determine whether IL-21 is capable of preventing tumour-mediated FOXP3 induction in naïve T cells, we repeated our naïve T cell stimulations in the presence of 50% cancer cell supernatants, alone or with recombinant human IL-21. Similarly to TGFβ blockade, IL-21 was able to potently inhibit baseline FOXP3 induction in naïve T cells (Fig. 5a) and FOXP3 induction as a result of the addition of cancer cell supernatants (Fig. 5a, b). Accordingly, the addition of IL-21 to these cultures also counteracted the marked inhibition of T cell proliferation observed in the presence of supernatants from cell lines representing colon and lung cancers (Fig. 5c).

**Fig. 6:**
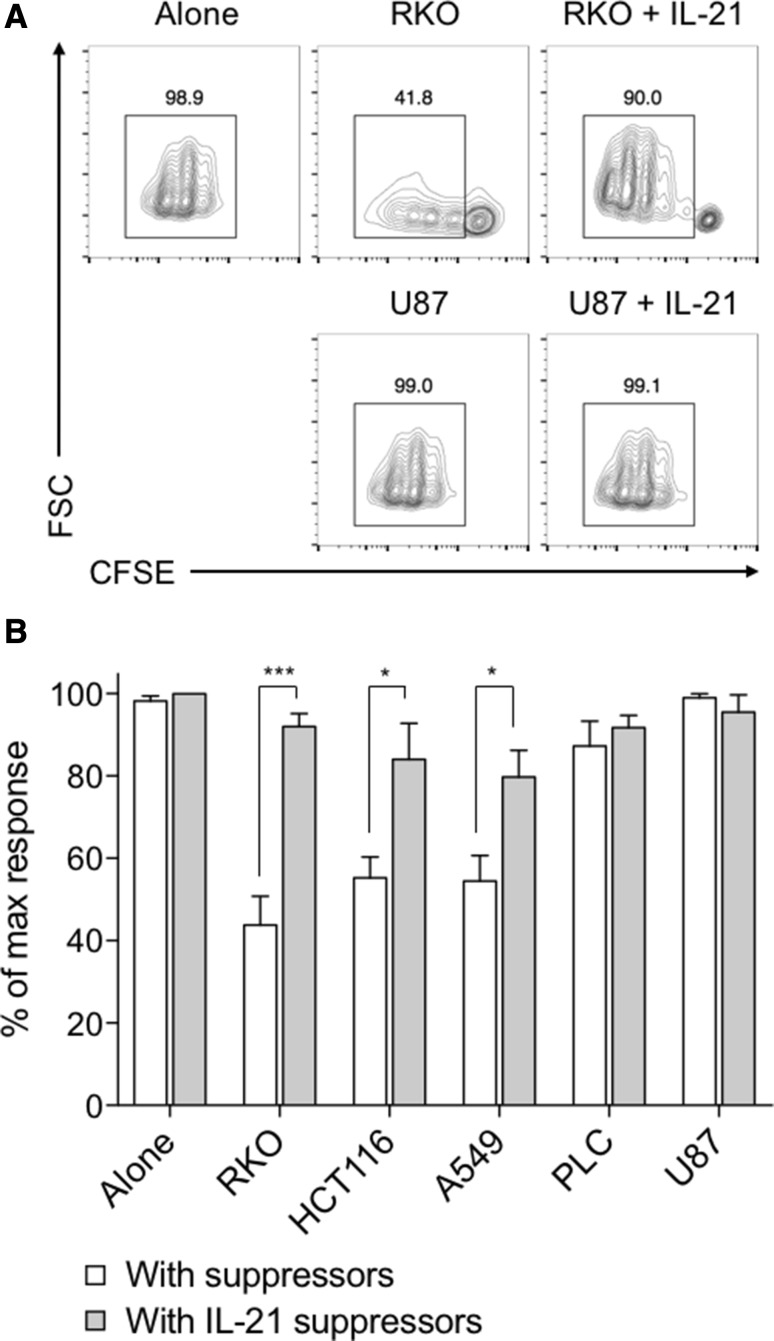
Cancer-mediated suppressor phenotype is maintained during subsequent T cell responses, and is counteracted by IL-21. **a** 2.5 × 10^4^ CD45RA+ CD4+ T cells from human peripheral blood were stimulated with anti-CD3/28 antibody-coated beads (1:1 ratio) alone, with either 50% culture supernatant from the indicated cell lines or 200 ng/mL IL-21, or both. After 5 days these suppressor cells were re-cultured with 2.5 × 10^4^ CFSE-labelled CD45RA+ CD4+ T cells from the same donor at a 2:1 ratio (suppressors to naïve cells), with anti-CD3/28 antibody-coated beads at a 1:1 ratio. Representative contour plots show CFSE dilution for naïve T cells cultured with RKO or U87 suppressors alone, or with these supernatants and IL-21. **b** Histogram shows collated CFSE+ CD4+ cell counts when cultured with suppressors derived from the indicated cancer cell supernatant and IL-21 cultures, expressed as a proportion of the control naïve T cell count in the absence of cancer supernatants. Data are representative of 4 independent experiments. *P < 0.05; **P < 0.01; ***P < 0.001

We next considered whether in the previous assay IL-21 might be directly promoting T cell proliferation, rather than indirectly promoting T cell responses by limiting FOXP3 induction. To address this, we repeated the cancer supernatant and IL-21 cultures described in Fig. 5, re-isolated the resultant T cells after 5 days, and assessed their ability to suppress CFSE-labelled naïve T cells from the same donor. Because IL-21 was not added to the second T cell cultures, any increase in T cell proliferation observed could only be mediated indirectly by IL-21 signalling to the suppressor population in the first-round cultures. This assay also had the advantage of allowing us to determine whether cancer-mediated acquisition of Treg characteristics was a stable phenotype. In these experiments, we observed inhibition of T cell responses consistent with the level of FOXP3 expression induced in the suppressor population by cancer cell supernatants (Fig. 6a, b). In accordance with its ability to inhibit FOXP3 expression, we observed significant restoration in T cell proliferation and total cell counts when suppressor populations were pre-incubated with IL-21.

## Discussion

Although the link between cancers and Treg induction has been previously investigated in murine studies [23], it has not been conclusively demonstrated in human cancers, wherein conventional CD4 T cells have been used, consisting of both naïve and memory populations [24, 25]. Thus, there has until now remained doubt over whether increased expression of FOXP3 in human studies is due to the expansion of pre-existing Treg populations, rather than de novo Treg generation. By sorting CD45RA+ naïve CD4 T cells in this study, our data reveal that a variety of cancers are able to directly mediate the differentiation of induced Treg from naïve human CD4 T cells.

Previous studies of FOXP3 induction in human CD4 T cells have found that this does not necessarily confer immunosuppressive capacity, as FOXP3 is transiently expressed during the activation of non-suppressive effector cells [26–28]. Indeed, our own data show that during stimulations of naïve T cells in the absence of any cancer cell supernatant, around 30% of T cells upregulate FOXP3 expression. In agreement with previous studies, these T cells were incapable of suppressing T cell responses in subsequent cultures, suggesting that they had not acquired an immunoregulatory phenotype. In contrast, addition of cancer cell supernatants further increased FOXP3 expression in naïve T cells, and this increased expression correlated not only with T cell inhibition in the same cultures, but in subsequent cultures with freshly isolated naïve T cells.

It has long been known that exogenous TGFβ has the ability to induce Treg differentiation from naïve CD4 T cells [21, 22], and it has been suggested in a number of murine studies that this mechanism might be important during tumour-mediated Treg induction [29–31]. Indeed, in this study we found that all of the cancer cell lines studied expressed TGFβ1 to some extent. RKO cells were found to induce the greatest expression levels of FOXP3 in naïve CD4 T cells, and blockade of TGFβ abrogated this effect, confirming the importance of this cytokine for tumour-mediated Treg induction in humans. Accordingly, U87 supernatants were found to contain significantly lower levels of both active and total TGFβ1 than RKO supernatants, and did not induce FOXP3 expression or inhibit T cell proliferation. Previous studies of U87 supernatants have suggested an ability to induce FOXP3 expression, although these data were obtained using conventional T cells rather than purified naïve T cells [24]. Interestingly, despite differences in experimental approach, FOXP3 induction appeared to be transient in this study and was not observed at day five, in agreement with our own data. Instability of FOXP3 induced by U87 supernatants would seem at odds with the demonstrated increased intratumoural Treg fractions found in glioma patients [2–4, 8]. However, it is important to note that some of these Treg might well be recruited natural Treg and that FOXP3-inducing factors within the tumour microenvironment would be continually expressed, and would, therefore, likely be sufficient to maintain stable FOXP3 expression in this setting.

The application of cytokines for cancer therapy has been extensively investigated and IL-2 has traditionally been thought to hold the greatest promise, stemming from its known capacity as a T cell growth factor. However, the discovery that IL-2 is critical for Treg survival and function [32–34] has raised serious concerns over its suitability. Indeed, IL-2 administration has been shown to increase Treg numbers in cancer patients and in murine tumour models [35, 36]. These undesirable traits have led to a renewed interest in cytokines capable of selectively promoting effector T cell responses, preferably whilst simultaneously inhibiting Treg-mediated immunosuppression. In these respects, IL-21 appears to be a far more appropriate candidate, not least because its administration to CD4 T cells drives their own responses and inhibits their ability to produce IL-2, depriving Treg cells of a non-redundant survival factor [15]. Unsurprisingly, a number of clinical trials of IL-21 administration in cancer therapy have now been initiated, either as a monotherapy or in combination with other agents [37].

In this study, we have assessed whether IL-21 is able to influence Treg induction in the setting of tumour-derived immunosuppressive factors. In this context, IL-21 acts a critical opposing force, specifically limiting tumour-mediated FOXP3 induction and preventing suppression of the naïve T cell response. As such, these results confirm an important additional role for IL-21 in promoting anti-tumour immunity. Thus, IL-21 should be effective regardless of whether intra-tumoural Treg are actively recruited natural Treg, in which case it would target their survival and function, or are induced within the tumour microenvironment, in which case it would target FOXP3 expression in naïve T cells. This two-pronged approach is, therefore, highly desirable, as it increases the likelihood of more completely restoring anti-tumour effector T cell responses. Our data also suggest that IL-21 could be beneficial as a therapeutic agent across a variety of cancers, due to its ability to inhibit FOXP3 induction across all the cancer cell lines tested.

## Abbreviations

CAR: Chimeric antigen receptor
CFSE: Carboxyfluorescein succinimidyl ester
CTLA-4: Cytotoxic T lymphocyte associated protein 4
FOXP3: Forkhead box P3
IL-21: Interleukin 21
TGFβ: Transforming growth factorβ
Treg: Regulatory T cell
